# Quantifying and Modeling Coordination and Coherence in Pedestrian Groups

**DOI:** 10.3389/fpsyg.2017.00949

**Published:** 2017-06-28

**Authors:** Adam W. Kiefer, Kevin Rio, Stéphane Bonneaud, Ashley Walton, William H. Warren

**Affiliations:** ^1^Department of Cognitive, Linguistic and Psychological Sciences, Brown UniversityProvidence, RI, United States; ^2^Division of Sports Medicine, Cincinnati Children's Hospital Medical CenterCincinnati, OH, United States; ^3^Department of Pediatrics, College of Medicine, University of CincinnatiCincinnati, OH, United States; ^4^Center for Cognition, Action and Perception, Department of Psychology, University of CincinnatiCincinnati, OH, United States

**Keywords:** group locomotion, group coordination, cross-recurrence quantification, principal components analysis

## Abstract

Coherent collective behavior emerges from local interactions between individuals that generate group dynamics. An outstanding question is how to quantify group coordination of non-rhythmic behavior, in order to understand the nature of these dynamics at both a local and global level. We investigate this problem in the context of a small group of four pedestrians walking to a goal, treating their speed, and heading as behavioral variables. To measure the local coordination between pairs of pedestrians, we employ cross-correlation to estimate coupling strength and cross-recurrence quantification (CRQ) analysis to estimate dynamic stability. When compared to reshuffled virtual control groups, the results indicate lower-dimensional behavior and a stronger, more stable coupling of walking speed in real groups. There were no differences in heading alignment observed between the real and virtual groups, due to the common goal. By modeling the local speed coupling, we can simulate coordination at the dyad and group levels. The findings demonstrate spontaneous coordination in pedestrian groups that gives rise to coherent global behavior. They also offer a methodological approach for investigating group dynamics in more complex settings.

## Introduction

Collective behavior in humans and other animals is thought to arise from local interactions between individuals that are coupled by sensory information. This coupling may be modulated by factors such as environmental context (e.g., presence of predators, food sources), motivation (e.g., metabolic state, goals), and cognitive or social constraints (e.g., strategies, group membership, dominance relations). To understand the emergence of collective behavior, researchers must characterize both the local coupling between individuals and the global patterns of coordination. Such an approach calls for a set of analytic tools that can quantify the degree and stability of spatio-temporal coordination at both the individual and collective levels. The purpose of this paper is to investigate coordination in human collective behavior, beginning with the analysis of local and global coordination in small pedestrian groups.

By way of introduction, consider the flocking behavior of a murmuration of starlings. Each bird is visually coupled to nearby neighbors, and this local coupling influences an individual's behavior in accordance with a particular set of “rules;” we call them control laws to emphasize their continuous dynamical as opposed to logical form. These local interactions give rise to coordinated behavior between neighbors, which in turn feeds back to involve more individuals, so the coordination pattern propagates through the flock. The end result is a self-organized pattern of global motion that emerges from local interactions. The exact nature of the control laws that govern these local interactions and how they generate coherent flocking behavior is an active area of research (Ballerini et al., 2008; Cavagna et al., 2010; Hildenbrandt et al., 2010; Lukeman et al., 2010).

It is difficult to infer the local control laws based solely on the observed global behavior, however. An important theoretical result is that different sets of interaction rules can generate the same pattern of coherent flocking (Vicsek and Zafeiris, 2012); thus, the local control laws are underdetermined by analysis of the global behavior. This finding implies that direct experimental study of interactions between individuals is required to model the control laws, which can then be used to simulate coordination patterns. Therefore, a complete account of collective behavior demands an approach that combines a local-to-global (bottom-up) perspective, in which empirically-grounded control laws are used to predict global behavior, and a global-to-local (top-down) perspective, in which measurements on global behavior are analyzed and compared with the predictions (Sumpter et al., 2012).

We are pursuing this dual approach to understand the collective behavior of human crowds. The program of research includes characterizing the control laws by which visual information guides locomotion, a pedestrian model that generates locomotor trajectories, and multi-agent simulations of the emergent crowd dynamics. Warren (2006) proposed a behavioral dynamics framework that aims to characterize how stable low-dimensional behavior emerges on-line from the interactions between an agent and its environment. Goal-directed behavior such as locomotion is regulated by perceptual information in accordance with task-specific control laws (Gibson, 1979; Warren et al., 2001; Warren and Fajen, 2004). Within this framework, Fajen and Warren (2003, 2007) and Warren and Fajen (2008) developed a pedestrian model that successfully characterizes locomotor behavior such as steering to stationary and moving goals, and avoiding stationary and moving obstacles. This model has recently been extended from agent-environment interactions to interactions between pairs of pedestrians (dyads), including pursuit and evasion, following, and walking side-by-side (Cohen et al., 2010; Bonneaud and Warren, 2012; Page and Warren, 2013; Rio et al., 2014).

In certain contexts, two pedestrians may have the goal of walking together, in which case they visually coordinate their velocity, i.e., walking speed and direction of travel (heading). During pedestrian following, Rio et al. (2014) found that the follower matches the leader's speed, independent of their interpersonal distance (1–3 m); this is accomplished by nulling the optical expansion of the leader (see also Lemercier et al., 2012; Bruneau et al., 2014). A similar speed-matching strategy was observed in side-by-side walking, with a similar coupling strength (Page and Warren, 2013). In addition, Dachner and Warren (2014) found that pedestrians match the walking direction of a neighbor, independent of interpersonal distance (1, 2, 4 m), with a comparable coupling strength in following and side-by-side walking. They recently proposed that speed and heading are jointly controlled by nulling both the optical expansion and the change in bearing direction of the leader (Dachner and Warren, 2017). These results indicate that pedestrian dyads utilize visual information to adopt a common speed and direction over a range of distances and positions.

This research has established a preliminary set of control laws that govern pedestrian interactions. An outstanding question is whether they scale from dyads to groups, and ultimately, can account for the self-organization of collective crowd behavior. Answering this question requires methods for quantifying the emergent patterns of coordination at both the local and global scales. This is a particularly difficult problem given that pedestrian locomotor trajectories are a continuously evolving, aperiodic behavior. Accordingly, it requires analysis tools that can identify the temporal pattern of non-rhythmic coordination between dyads at a local level, as well as group coherence at a global level.

As a first step, the system must be operationalized. In previous work, two behavioral variables have been used to describe a locomotor trajectory: (1) the agent's direction of heading (Φ), and (2) the agent's speed (*s*), which together define the agent's velocity in an allocentric coordinate frame. This operationalizes a pedestrian as having two degrees of freedom (DoF), which may be coupled between neighbors. Similarly, Riley et al. (2011) proposed that behavioral coordination between two agents arises from the coupling of their DoF. It is believed that agents couple the DoF of a system via shared information variables, so that the DoF directly regulate one another. Hence, the control of behavior at the level of the group emerges via functional, information-based linkages between the behavioral variables of individual agents. When framed in terms of behavioral dynamics, collective behavior can be considered a problem of informationally coupling the appropriate behavioral variables to yield a stable solution of the global behavioral dynamics. For the task of locomotion, each pedestrian is operationalized as a two DoF system with the state variables Φ and *s*. Each additional individual in a group of N pedestrians would add two more state variables to the collective system, so the total DoF = 2N. Thus, the state space of the system has 2N dimensions.

Once the behavioral variables are identified, the next step is to quantify the degree of coordination at the collective level. From a global perspective, the degree of coordination among a set of pedestrians would be reflected in a reduction of the effective DoF of the system to a value between 2N, such that all individuals move independently, and 2, such that all individuals move with the identical speed and direction. One way to measure the reduction in a system's DoF is to quantify the dimensional compression of the observed behavior. Principle Components Analysis (PCA) is a valuable tool in this regard (Riley et al., 2011). PCA can be used to identify collective variables, or principle components, based on the relations among observations in a high-dimensional state space (cf. Haken and Wunderlin, 1990). It also indexes the load magnitude of each state variable on the identified principle components, which can help uncover the coupling between behavioral variables. The strength of PCA is its ability to include many variables of a complex system in a single analysis and to provide an output that quantifies the degree of relation, or even coordination, between the component variables. Its limitation is that PCA is a linear analysis, and therefore assumes linear relations among the system's variables. PCA provides the first part of the analysis by quantifying group coherence at the global level.

At the local level, the next step is to quantify the degree of coordination between pairs of individuals in a group, to reveal the coupling strength as a function of variables such as neighbor distance and position. One approach is to compute the linear cross-correlation between the time series of speed (or heading) for two pedestrians. The limitation of this analysis is that it assumes that individuals are coupled at a single time-scale and that behavior is stationary (i.e., a constant delay). It therefore has limited utility in analyzing more complex systems, such as bidirectional coupling at multiple time-scales and non-stationary behavior that evolves over time.

Cross-recurrence quantification (CRQ), is well-suited to the latter type of data and has proven useful in analyzing interpersonal coordination (cf., Shockley et al., 2003; Richardson, D. C. et al., 2007; Ramenzoni et al., 2012). CRQ is a non-linear analysis that indexes repeating patterns in a pair of time series at multiple temporal scales (Webber and Zbilut, 1994; Shockley et al., 2002). In particular, the output measure “cross-maxline” (CML) has proven to be a reliable estimate of the temporal stability of coordination, associated with coupling strength, between two movements (Richardson, M. J. et al., 2007; Page and Warren, 2013). However, these local analyses are limited to a pairwise comparison of dyads in a group.

Finally, to determine whether a model of the local coupling can account for the observed patterns of coordination, agent-based simulation methods can be used to try and reproduce the data. In particular, we investigate the mechanism of coordination by testing whether our model of the local “rule” for speed matching, derived from data on pairs of pedestrians, generalizes to coordination in a group, and can explain the adoption of a common collective speed and heading.

**Figure 1:**
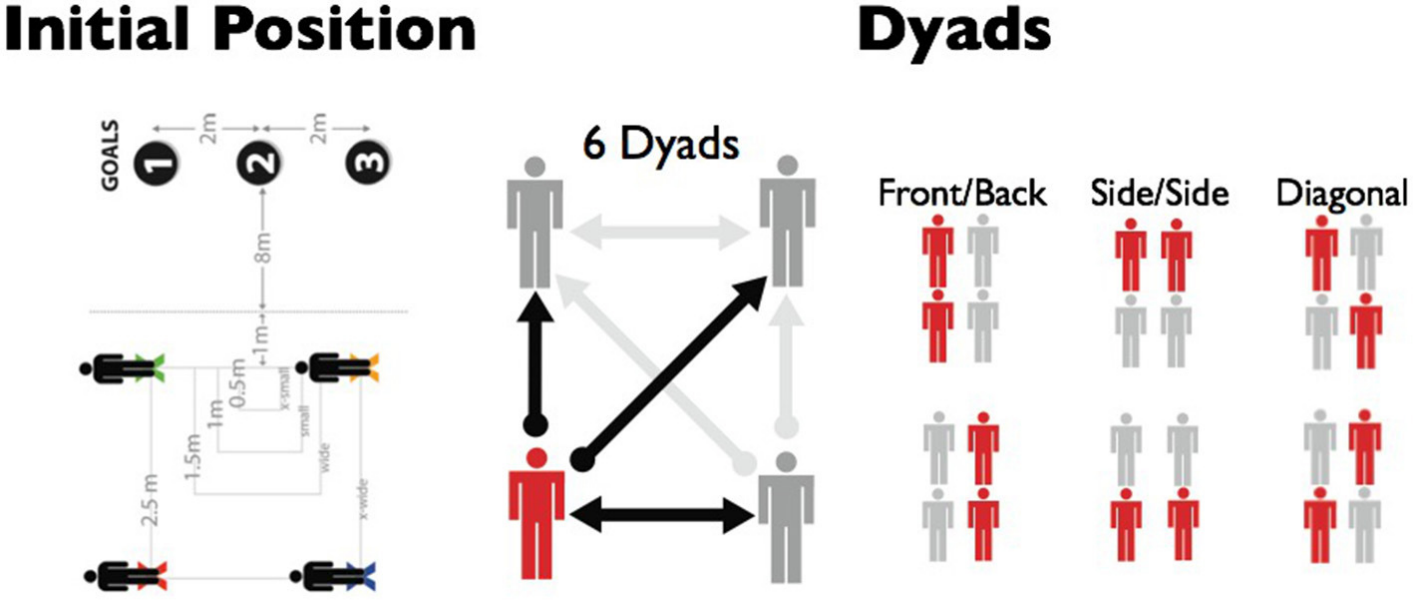
The four possible starting positions for each of the four possible starting densities (left). Note the dotted “trigger” line 1 m from the midpoint between the front two participants that represents when the experimenter “goal” command was given. The visual couplings of the six possible dyads (center) with double arrows indicating bi-directional vs. unidirectional (single arrow) coupling. The six dyads are highlighted in the right pane.

Our goal in the present paper is to measure the degree of coordination in pedestrian groups at the global and local levels, and to model the local coupling that generates such coordination. Establishing the emergence of coordinated behavior is prerequisite to modeling the informational control laws, characterizing the conditions for the emergence of such behavior, and eventually investigating the roles of other cognitive and social variables. In the present experiment, groups of four pedestrians walked toward one of three goals, while the group's initial density (interpersonal distance) was varied on each trial (see Figure 1). The role of density is important due to its potential contribution to self-organization: if coupling strength is distance-dependent, higher densities would create stronger local interactions and promote coherent crowd formation. Previous results have shown that, for an individual pedestrian, the coupling to obstacles decays exponentially with distance, asymptoting at 3–4 m (Fajen and Warren, 2003), but on the other hand, the coupling between pairs of pedestrians appears to be independent of distance, at least up to 3–4 m (Dachner and Warren, 2014; Rio et al., 2014). In the present experiment, we explored interpersonal distances of 0.5–2.5 m within groups of four people.

As described above, we analyzed two behavioral variables: the walking speed *s* and walking direction Φ for each agent. This resulted in a total of eight state variables, or DoF, for the four-agent system. To determine whether the observed coordination is a consequence of the informational coupling between individuals and is not due to other task constraints, we compared the real groups with virtual groups that were constructed by randomly sampling the same four pedestrians from four different trials. At the global level, we hypothesized that the real groups would exhibit dimensional compression in all conditions, compared to the virtual groups. We also investigated whether dimensionality would be reduced more in the higher density conditions. At the local level, we hypothesized that the coupling strength would be greater between real dyads than virtual dyads, and we asked whether it would increase as a function of group density. Finally, we tested whether Rio et al.'s (2014) speed-matching model generalizes to the observed speed coordination between individuals in a group and can explain the emergence of a common speed.

## Method

### Participants

Five groups of four participants (*N* = 20; M age 23.57 ± 0.93 years; 12 female, 8 male), students at Brown University, were compensated $15 for their participation. Participants had normal or corrected-to-normal vision and no history of cognitive deficits, lower extremity injury, or neuromuscular disorders that would inhibit normal locomotor activity. This study was carried out in accordance with the recommendations of the Brown University Institutional Review Board with written informed consent from all subjects. All subjects gave written informed consent in accordance with the Declaration of Helsinki. The protocol was approved by the Institutional Review Board.

### Materials and apparatus

The experiment was conducted in the VENLab at Brown University, a 12 × 14 m open room. The head position of each participant was tracked with a MicroTrax inertial tracker affixed atop a lightweight bicycle helmet on the head. Each tracker communicated with an IS-900 ultrasonic overhead grid tracking system (InterSense, Billerica MA, USA) and provided 6 DoF position (4 mm RMS error) and orientation (0.1° RMS error) data at 60 Hz. Three cardboard goal poles (~2 m tall and 0.5 m in diameter) were placed at an initial distance of 8 m from the “trigger line” for the front two participants, and spaced 2 m apart, with goal 2 straight ahead, goal 1 to the left, and goal 3 to the right (see Figure 1). Colored tape was used to mark four possible starting positions in a square configuration, with initial interpersonal spacing of 0.5, 1.0, 1.5, or 2.5 m on a side.

### Design and procedure

Each group completed eight trials in each of 12 conditions (see Figure 1), with four densities (interpersonal distances of 0.5, 1.0, 1.5, 2.5 m) crossed with three goal positions (left, straight, right). This resulted in a total of 96 trials, presented in a random order, in each experimental session. Goal position was manipulated in order to vary the heading direction between trials, and thus was not included as a factor in the statistical analyses.

At the beginning of each trial the four participants were randomly assigned to the four positions in the square configuration: (1) front right, (2) front left, (3) back right, or (4) back left (Figure 1). Once they were standing in the correct location, an experimenter gave a verbal “go” signal and the group began to walk straight ahead. As the last participant crossed a notional “trigger line” 1 m after the starting line, the experimenter gave a verbal command of goal 1, 2, or 3. The only instruction given to the participants was to walk to the specified goal at a comfortable pace without stopping. Participants were not told to stay together as a group or to maintain their initial configuration. Each trial lasted ~6–8 s.

### Data reduction and analysis

The tracking system recorded the medial-lateral and anterior-posterior head position (*x*- and *z*-coordinates, respectively) of each participant at a sampling rate of 60 Hz. The raw (unfiltered) position data were used to compute the participant's *s* and Φ from the displacement between successive samples, according to the following equations:

(1) $$s_{i}=\frac{{\left({\left(x_{i}-x_{i-1}\right)}^{2}+{\left(z_{i}-z_{i-1}\right)}^{2}\right)}^{0.5}}{\Delta t},$$

(2) $$\phi_{i}=\;{\text{tan}}^{-1}\left(\frac{x_{i}\;-\;x_{i-1}}{z_{i}\;-\;z_{i-1}}\right),$$

where *x*_*i*_ and *z*_*i*_ are the head position on the *i*th frame, in room coordinates. The Φ and *s* time series were used for all subsequent analyses.

#### Virtual group construction

Certain aspects of the procedure—such as a common goal, a simultaneous go signal, a simultaneous goal command, and walking at preferred speed—may have yielded correlations between participants that were not due to the visual coupling. To isolate the effect of the coupling from these task constraints, the data from real groups were compared with control data from constructed virtual groups that were not visually coupled. For each real group trial, a paired virtual group trial was created by randomly selecting a time series for the same four participants in the same condition, but from four different trials. Thus, all task constraints were matched, except that the participants in the virtual group were not perceptually coupled with each other. The four randomly selected time series were temporally aligned based on the goal command, and their lengths equated by cropping the beginning and/or end of the time series, to match the length of the shortest time series (a requirement of both PCA and CRQ analysis). This resulted in four randomly selected time series of equal length that were aligned by the goal command. Using these virtual groups as a control ensured that any significant coordination between participants was due to the perceptual coupling, not the task constraints.

#### Principal components analysis (PCA)

PCA identifies linear relationships within multi-dimensional datasets and then maps the original data into a newly defined space, with the principal components as its axes. The principal components represent the dataset's primary dimensions of variation, but do not necessarily map directly onto the original dimensions of the actual measurement. The end result is a representation of potentially new, important collective variables that best account for the variance within the observed system.

In the context of the present experiment, eight variables of interest representative of the 8 DoF of the observed system (i.e., Φ and *s* for each of the four participants in each group) were submitted to a single PCA. The data were normalized using a *z*-score transform prior to analysis. PCA was performed in *Matlab* using the *princomp* function and the results were examined in a similar fashion to Ramenzoni et al. (2012). First, the number of components that together account for 90% or more of the variance in the data set was determined. To investigate dimensional compression in the real vs. virtual group, a 4 × 2 mixed-model ANOVA was conducted on number of components, with initial density as a within-subjects factor and group (real vs. virtual) as a between-subjects factor, averaged across goal position. Next, the amount of variance accounted for by the first principal component (PC) in the real vs. virtual group was compared using an identical mixed-model ANOVA. The analysis was limited to the first two PCs because (a) the subsequent components were dependent on the first PC, and (b) the second PC provides additional context about the subsequent loadings. Greater variance accounted for by the first PC in the real group indicates dimensional compression, and thus greater coherence, in the visually coupled system. Finally, the mean correlation coefficient (*r*) for the loading of each behavioral variable on the first PC was examined to investigate which of the eight variables were most influential in characterizing the group's behavior. The *r*-values were transformed using a Fisher's *z*' transform and submitted to a 4 × 8 × 2 mixed-model ANOVA with initial density and agent position as within-subjects factors, and group as a between-subjects factor, again averaged across goal position for PC1. The aim of this analysis was to examine whether the speed or heading of an agent in a particular position more strongly influenced the group's behavior and whether this influence depended on density.

#### Cross-correlations

At the local level, linear cross-correlation was used to measure the strength of the relation and the time delay between pairs of Φ time series (and, separately, pairs of *s* time series) for each of the six dyads in a group (illustrated in Figure 1, right). On each trial, the cross-correlation between the two time series for each dyad was computed, varying the time delay from −2,000 to +2,000 ms (where positive delays imply that the back participant lags behind the front participant, or the left participant lags behind the right participant in side-by-side dyads). For statistical comparisons, mean *r*-values for each participant were computed using Fisher's *z* transform to correct for non-normality and submitted to a 4 × 6 × 2 mixed-model ANOVA (density × dyad × group); the mean *z*-values were transformed back into the mean *r*-values reported below. A similar ANOVA was performed on the optimal delay for each pair of time series.

#### Cross-recurrence quantification (CRQ)

**Figure 2:**
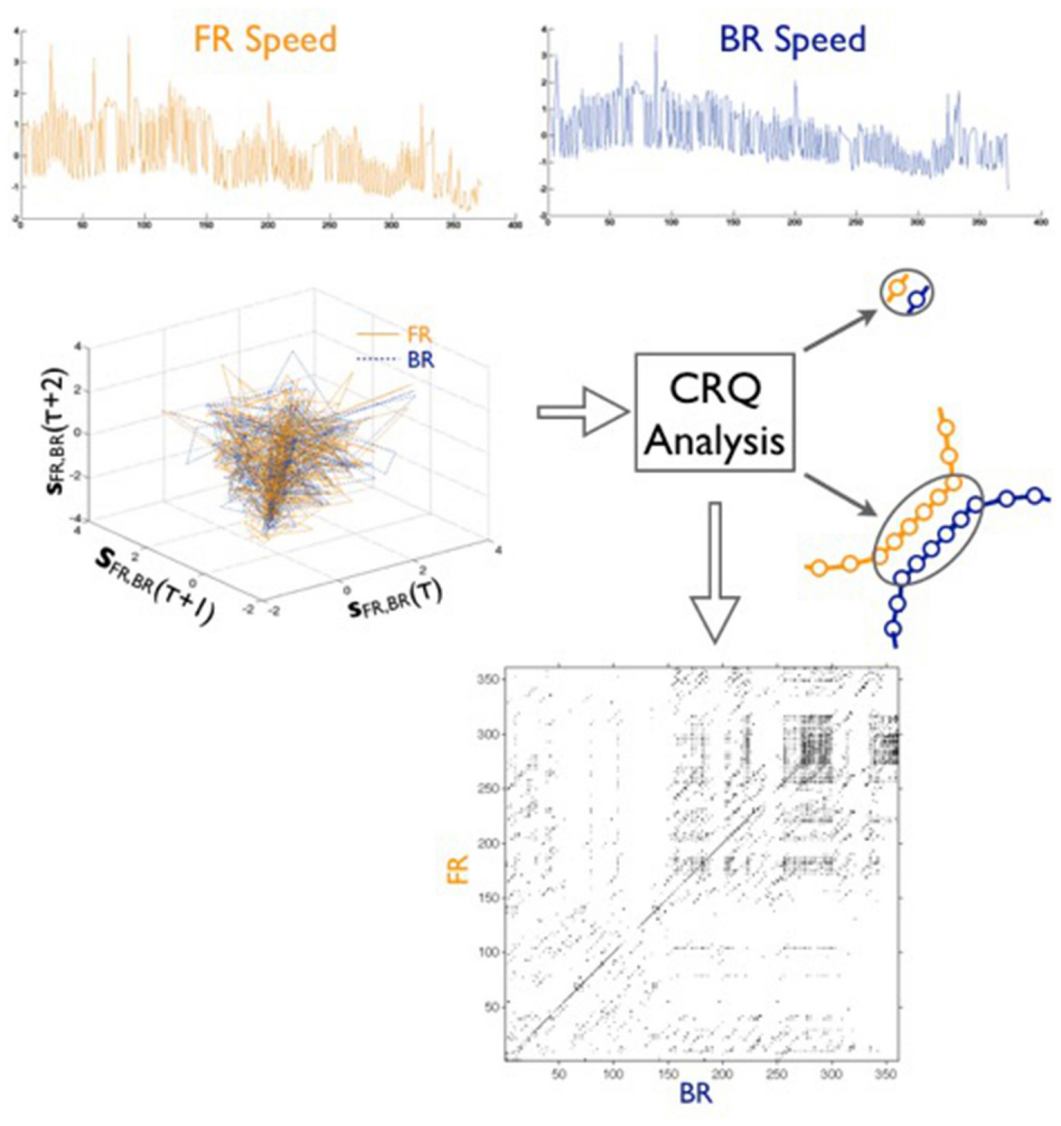
A schematic of the steps in the CRQ analysis. For each trial, the speed time series of one agent (FR = top left) and a second agent (BR = top right) are unfolded separately into a shared reconstructed phase space via time-delayed copies of each measured time series, denoted as **s**_FR, BR_ (center, left). Recurrent points within a given radius and strings of recurrent points are identified with respect to each point in phase space and represented in a cross-recurrence plot (center, right), in which each axis represents the **s**_FR_ and **s**_BR_ time series at each time step. Each pixel indicates a recurrent point on a recurrence plot (bottom), and the diagonal line structures indicate the length of a string of recurrent points, or the co-evolution of the two time series at different time delays. The longest diagonal line, cross-maxline (CML), was computed for each dyad in the group.

A non-linear, two-dimensional CRQ analysis was used to quantify the time-correlated activity between pairs of Φ time series (and pairs of *s* time series) for each dyad in a group. Referring to Figure 2, a CRQ analysis is conducted by first embedding the pair of normalized time series in a multidimensional, time-delayed phase space (see Webber and Zbilut, 1994; Shockley et al., 2002; Marwan et al., 2007). Because not all variables that make up the behavior in a dynamical system are necessarily knowable *a priori*, phase space reconstruction allows for the behavior of these potentially “hidden” variables in the dynamical system to be evaluated via their interaction with, or influence on, the known variable (in this case the Φ or *s* time series). Hence, the structure of the reconstructed phase space can reveal the underlying dynamics of the dynamical system as a whole. Specifically, the “neighborliness” of points within some tolerance or radius in phase space can indicate recurrent points in the two time series. These points represent states in one time series that closely correspond to previous, current or future states in the other time series, and can illustrate behavioral patterns of coordination in the observed system. The recurrent points are identified and represented in a cross-recurrence plot (see Figure 2, bottom), from which a suite of measures can be computed to quantify these patterns (see Shockley et al., 2002; Marwan et al., 2007 for a review of analysis procedures).

The present experiment focused on cross-maxline (CML): specifically, the longest diagonal line of consecutive recurrent points on a cross-recurrence plot. This provides a measure of the longest time interval that the heading (or speed) of two participants was coupled (i.e., the two participants maintained the same direction of travel or walking speed, as specified by a predetermined threshold viz. radius) during a given trial, and this interval could occur at any point during a given trial. CML is known to be sensitive to the temporal stability of coordination between two time series, associated with coupling strength. The parameters used for CRQ were as follows: for Φ, embedding dimension = 6; delay = 4 data points; radius within which points are counted as recurrent = 0.7% of the actual distance separating points in reconstructed phase space, and for *s* embedding dimension = 5; delay = 3 data points; radius within which points are counted as recurrent = 1.0% of the actual distance separating points in reconstructed phase space.

## Results

### Principal components analysis

**Figure 3:**
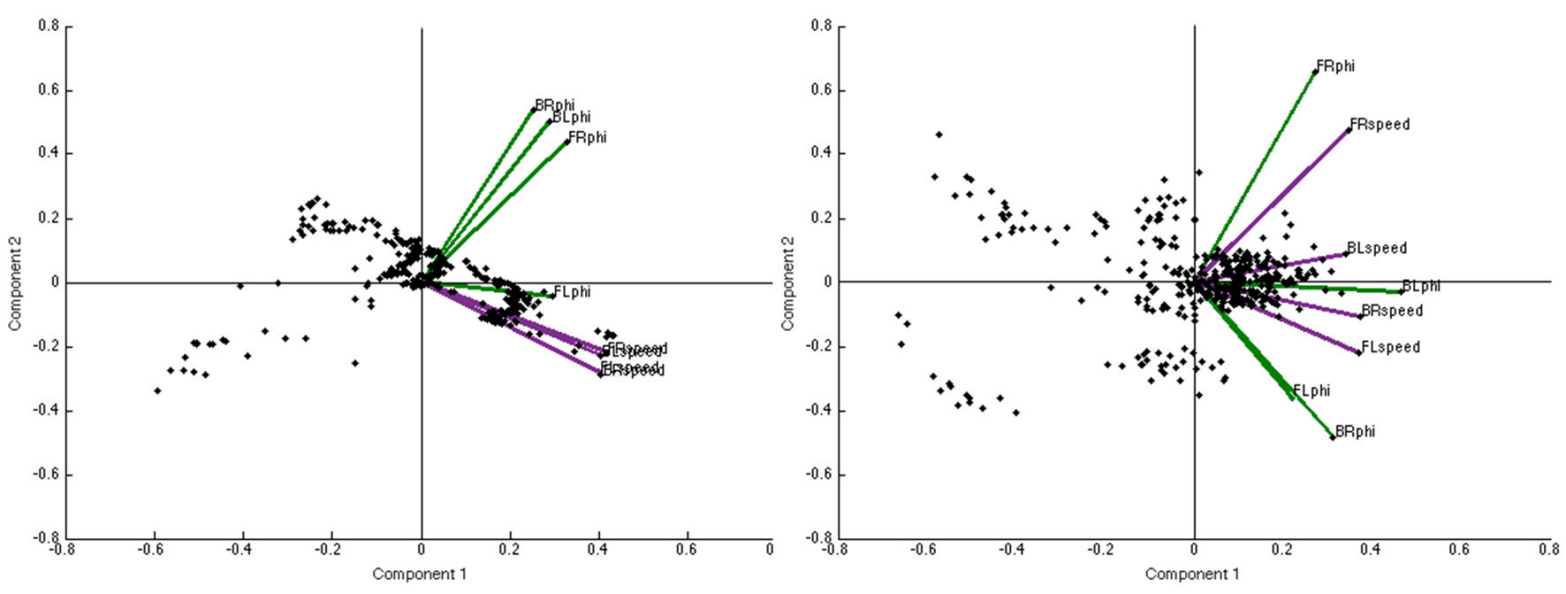
Sample biplots of PC coefficients for a real group trial **(Left)** and a virtual group trial **(Right)**. These offer a representation of both the observations and variables of PC coefficients with the proximity of each trend line corresponding to the coefficient values for that particular variable.

See Figure 3 for sample biplots—a representation of both the observations and variables—of PC coefficients for a real group (left panel) and a virtual group (right panel). The clustering of the speed variables along the positive x axis of the real group (left) indicates a consistent, positive loading of those variables on PC1, as contrasted with the virtual group (right) where the variables exhibit greater variance around both the positive x (PC1) and positive y (PC2) axes.

#### Number of components

**Figure 4:**
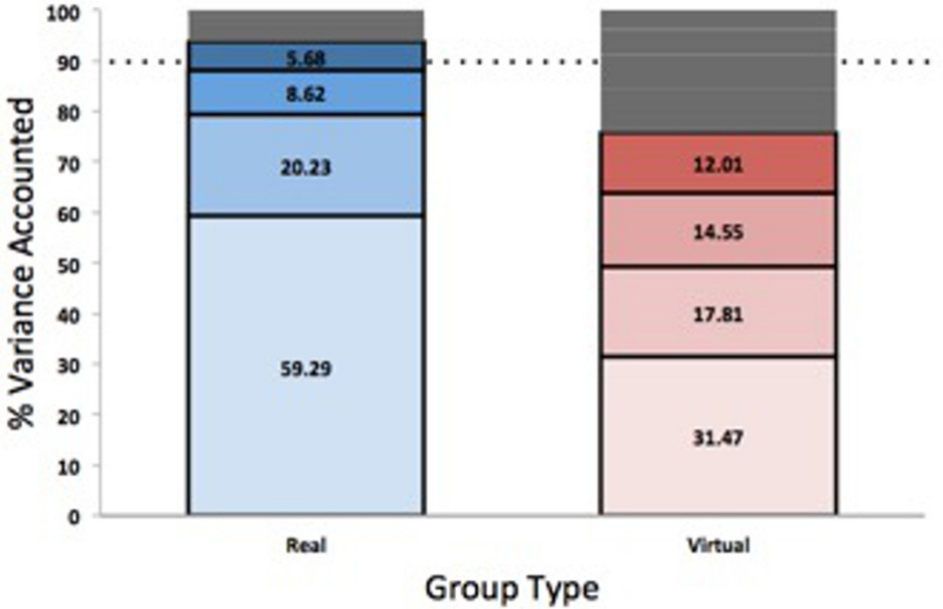
The amount of variance accounted for by each component beginning with PC1.

The number of components required to account for 90% of the variance was significantly lower in real groups (*M* = 3.61 ± 0.12) compared to virtual groups (*M* = 6.18 ± 0.07), *F*_(1, 8)_ = 583.95, *p* < 0.001, η^2^ = 0.99 (see Figure 4). Thus, the external task constraints appear to reduce the group DoF from 8.0 to 6.18, and the perceptual coupling between participants further reduced the DoF to 3.61, consistent with the emergence of global coordination. There was a significant interaction between group and density, *F*_(3, 24)_ = 3.46, *p* = 0.032, η^2^ = 0.30; *post-hoc* tests revealed that this was driven by the group difference with the real groups exhibiting a lower number of components needing to account for 90% of the variance. No other main effects of dyad or density were found (*p* > 0.05).

#### PC1

The first principal component accounted for significantly more variance in real groups (*M* = 59.29% ± 0.79) than in virtual groups (*M* = 31.47% ± 0.45), *F*_(1, 8)_ = 142.60, *p* < 0.001, η^2^ = 0.95. This result confirms dimensional compression in group behavior due to the visual coupling. There was also no main effect of initial density on the variance accounted for by PC 1, and no interactions.

#### Contribution of variables to PC1

**Figure 5:**
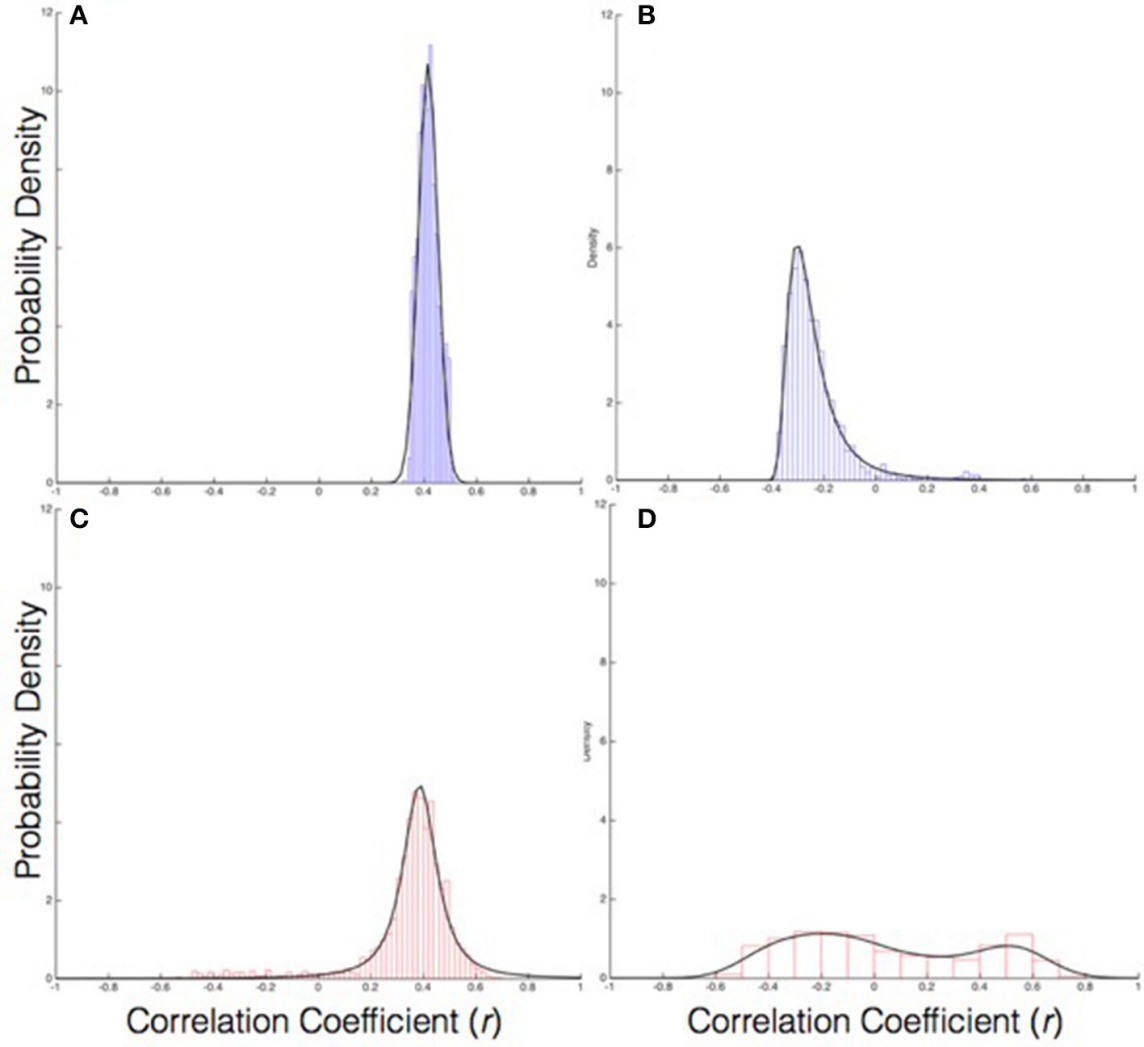
The probability distribution of PC coefficients (r) for the speed variable: **(A)** Real group PC1, **(B)** Real group PC2, **(C)** Virtual group PC1, **(D)** Virtual group PC2.

**Figure 6:**
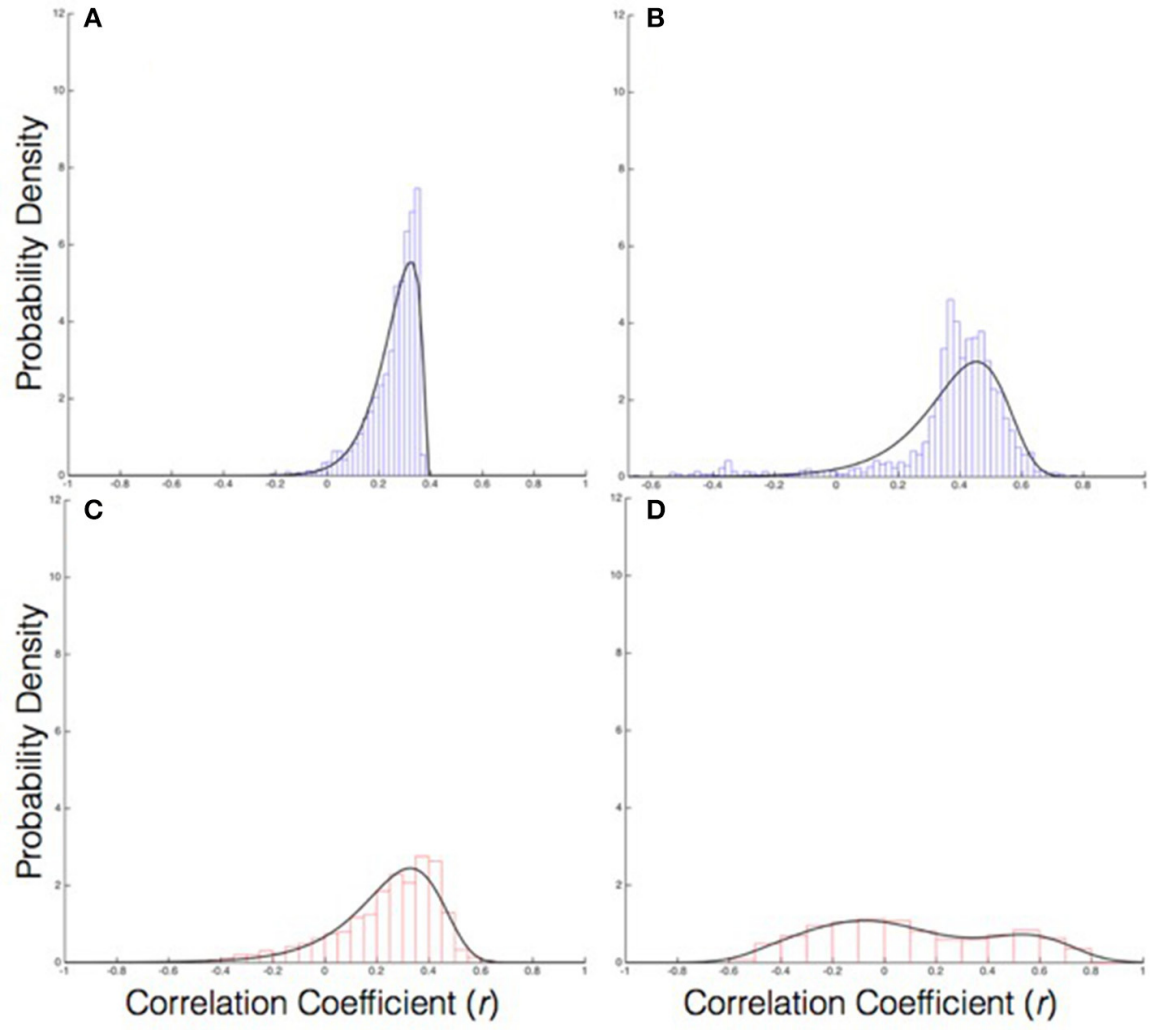
The probability distribution of PC coefficients (r) for the heading variable: **(A)** Real group PC1, **(B)** Real group PC2, **(C)** Virtual group PC1, **(D)** Virtual group PC2.

The composition of the first principal component was further examined to determine the relative contribution of each of the eight behavioral variables, by computing the loading (*r*) of each variable on PC1. Overall, the *s* and Φ variables for all agent positions in the real group exhibited a stronger correlation with PC1 than they did in the virtual group (*M* = 0.36 ± 0.006 and *M* = 0.31 ± 0.008), *F*_(1, 8)_ = 31.23, *p* < 0.001, η^2^ = 0.78, suggesting that the behavior of real groups was more coherent than that of virtual groups. There was also a main effect of position, *F*_(7, 56)_ = 52.27, *p* = 0.000, η^2^ = 0.867. Follow-up *t*-tests (Bonferroni corrected *p* ≤ 0.01) indicated that across all agent positions, the *s* variable was more strongly correlated with PC1 in the real groups than in the virtual groups (all *p* < 0.001), whereas there were no group differences for the Φ variable (all *p* > 0.01). Within the real groups, the *s* variable had a higher correlation than the Φ variable (*p* < 0.001), whereas in the virtual groups, *s* and Φ did not significantly differ (all *p* >0.01). Greater group coordination was, therefore, primarily due to the visual coupling of walking speed; in contrast, individual headings were generally aligned whether or not participants were visually coupled, presumably due to the presence of a common goal. See Figures 5A,C for the distribution of correlation coefficients for the loading of speed on PC1 in the real and virtual groups, and Figures 6A,C for the corresponding distributions for heading. The descriptive values of skewness, kurtosis and variance for all coefficients loading on PC1 appears in Table 1.

**Table 1:** Descriptive properties of the Φ and *s* PC coefficient distributions for PC1 and PC2.

**Variable**	**Real Group**			**Virtual Group**		
	**Skewness**	**Kurtosis**	**Variance**	**Skewness**	**Kurtosis**	**Variance**
Φ–PC1	−1.44	5.18	0.01	−1.03	3.73	0.04
Φ–PC2	−2.35	10.02	0.04	0.21	1.95	0.12
***s***–PC1	0.33	2.45	0.01	−2.39	9.94	0.03
***s***–PC2	2.58	12.62	0.01	0.28	1.78	0.12

#### PC2

The second principal component was also examined to determine the amount of variance accounted for in each group. The results indicated that PC2 accounted for significantly more variance in real groups (*M* = 20.23% ± 0.68) compared to virtual groups (*M* = 17.81% ± 0.68), *F*_(1, 8)_ = 21.88, *p* = 0.002, η^2^ = 0.73. There was no main effect of density nor significant interaction effects (*p* > 0.05).

#### Contribution of variables to PC2

Negative correlation coefficients were prevalent for PC2. Because of this, analyses were limited to qualitative observations and descriptive characteristics of the distribution of coefficients and skewness, kurtosis, and variance. The distribution of correlation coefficients for speed as it loaded on PC2 exhibited a negatively skewed, unimodal distribution for the real group compared to a somewhat biomodal distribution with almost no skew in the virtual group (See Figures 5B,D for the distribution of coefficients for speed in the real and virtual group, respectively). Similarly, the distribution for heading as it loaded on PC2 exhibited a bimodal distribution with less skew than the virtual group (see Figures 6B,D for the distribution of coefficients for heading). See Table 1 for skewness, kurtosis, and variance descriptive values for all coefficients loading on PC2.

### Cross-correlations

#### Speed *(s)*

ANOVA on transformed *r* revealed a main effect of group, *F*_(1, 8)_ = 57.76, *p* < 0.001, η^2^ = 0.88, such that the real group was more strongly coupled than the virtual group (*M* = 0.832 ± 0.021 vs. 0.358 ± 0.166, respectively). There was also a significant group × density × dyad interaction, *F*_(9, 72)_ = 2.88, *p* = 0.006, η^2^ = 0.22. Bonferroni-corrected *t*-tests revealed that all real group dyads had a significantly higher correlation compared to the virtual group dyads (*p* < 0.001). No other comparisons were significantly different. ANOVA on the optimal delay revealed a group × dyad interaction, *F*_(3, 24)_ = 3.02, *p* = 0.05, η^2^ = 0.22, with follow-up tests indicating that the optimal delay for the real group back side-to-side dyad was significantly lower (*M* = 0.00 ± 0.00 s) compared to the corresponding virtual group dyad (*M* = 0.04 ± 0.07 s). No other significant differences were found with respect to group, density or dyad.

#### Heading (Φ)

ANOVA on *r* revealed no significant effects of group, dyad, density or interactions between/among these factors. ANOVA on delay revealed a significant main effect of dyad, *F*_(3, 24)_ = 3.16, *p* = 0.04, η^2^ = 0.24; however, Bonferroni corrected *post-hoc* tests did not reveal any significant differences between the various dyads. No other effects of group, density or dyad were observed. As mentioned above, because all participants turned to walk to a common goal, their heading directions were highly correlated in the virtual group as well as the real group.

### Cross recurrence quantification

#### Cross-maxline for *s*

**Figure 7:**
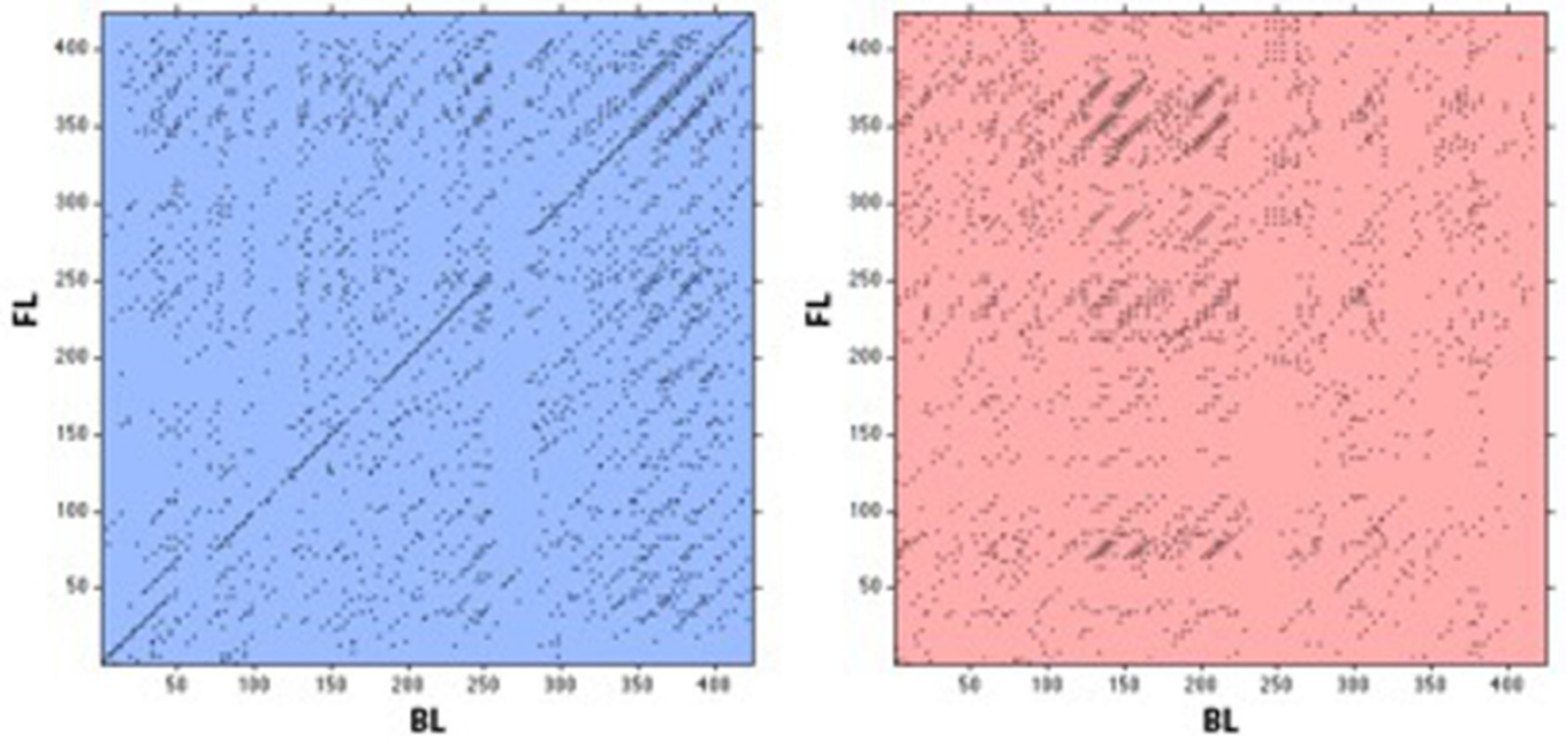
Sample cross-recurrence plots for speed time series from a real **(Left)** and a virtual **(Right)** leader-follower dyad. Note the presence of a main diagonal line (i.e., line of synchronization) and the additional diagonal lines that are visible in the cross-recurrence plot for the real dyad. These are indicative of a temporally stable speed coupling between agents.

**Figure 8:**
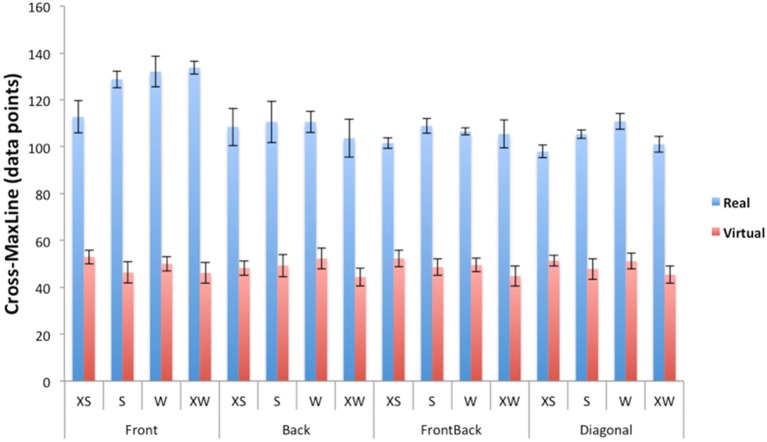
CML values for real compared to virtual groups for each of 4 densities and 6 dyads. All real group conditions were significantly greater than virtual group conditions (Bonferroni corrected *p* ≤ 0.01). XS = 0.5 m apart, S = 1 m apart, W = 1.5 m apart, XW = 2.5 m apart.

Representative cross-recurrence plots for speed from a trial with a real dyad (Figure 7, left) and virtual dyad (Figure 7, right). Prior to inferential analyses a log10 transform was conducted to correct for positive skewness in the data. A significant main effect of group was observed on CML, *F*_(1, 8)_ = 87.90, *p* < 0.001, η^2^ = 0.917. Specifically, the real group exhibited an average CML (*M* = 111.13 ± 10.92 samples) more than twice as long as the virtual group (*M* = 48.78 ± 2.79 samples), irrespective of dyad or initial density. This result demonstrates that the speed coupling is significantly more stable in the real than the virtual groups. There were no main effects of density or dyad, but a significant density × dyad × group interaction was found, *F*_(9, 72)_ = 3.16, *p* = 0.003, η^2^ = 0.283. Follow-up *t*-tests (Bonferroni corrected *p* ≤ 0.01) indicated that the real groups were more strongly coupled than the virtual groups for all densities and dyads, but no other effects were significant (see Figure 8). These results imply that the speed coupling is equally stable at high and low densities, and for leader-follower and side-by-side dyads.

## Modeling

Given that speed coordination was significantly greater in real than virtual groups, whereas heading coordination was not, we proceeded to simulate speed coordination in real groups based on Rio et al.'s (2014) model of the local coupling. A dyad was simulated by using the time series of speed for one participant (the “leader”) as input, and computing the time series of acceleration for a model “follower,” according to Equation (1):

(3) $${\ddot{x}}_{f}=c\cdot [{\dot{x}}_{l}-{\dot{x}}_{f}]$$

where $\dot{x}$_*l*_ is the leader's speed, $\dot{x}$_*f*_ is the follower's speed, and *c* is a gain parameter. We adopted c = 1.87, the best-fit parameter value from Rio et al. (2014), and the initial speeds of the leader and follower were zero. The simulation was evaluated by comparing the time series of the model “follower” with that of the human “follower.”

Simulations were performed for each dyad on each trial. The six dyads were classified into three dyad types: front-back, side-by-side, and diagonal (see Figure 1). Front-back dyads were symmetrical relative to the group's walking direction, so they were analyzed together; the same held for diagonal dyads. By contrast, the side-by-side dyads were fundamentally different from one another; pedestrians in the front side-by-side dyad were visually coupled only to each other, while those in the back side-by-side dyad could potentially receive visual information from all three neighbors in the group. For this reason, the front side-by-side and back side-by-side dyads were analyzed separately.

For front-back and diagonal dyads, the front participant served as the “leader” and the back participant as the modeled “follower;” side-by-side dyads were simulated twice, with the left (right) participant as the “leader” and the right (left) participant as the modeled “follower.” Performance was evaluated by computing the correlation coefficient (Pearson's *r*) between the simulated “follower” time-series and the observed time-series of the human “follower” on each trial; root-mean-squared-error (RMSE) between the two time series was also analyzed.

### Simulations of speed coordination

**Figure 9:**
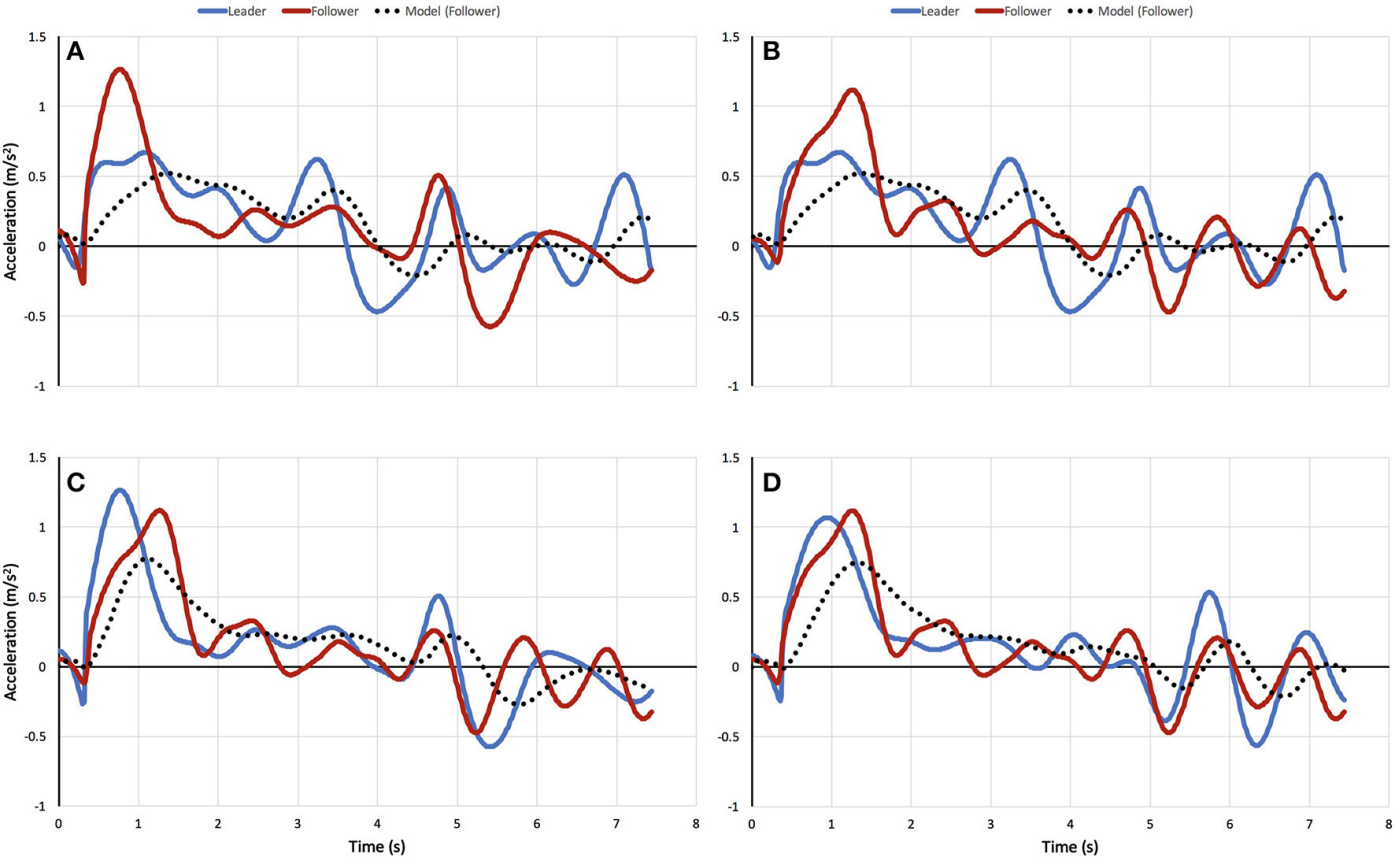
Sample time series of the simulated and observed “follower” acceleration (both in red), together with the observed “leader” acceleration (in blue), for front side-to-side **(A)**, leader-follower diagonal **(B)**, leader-follower **(C)**, and back side-to-side **(D)** dyads.

**Figure 10:**
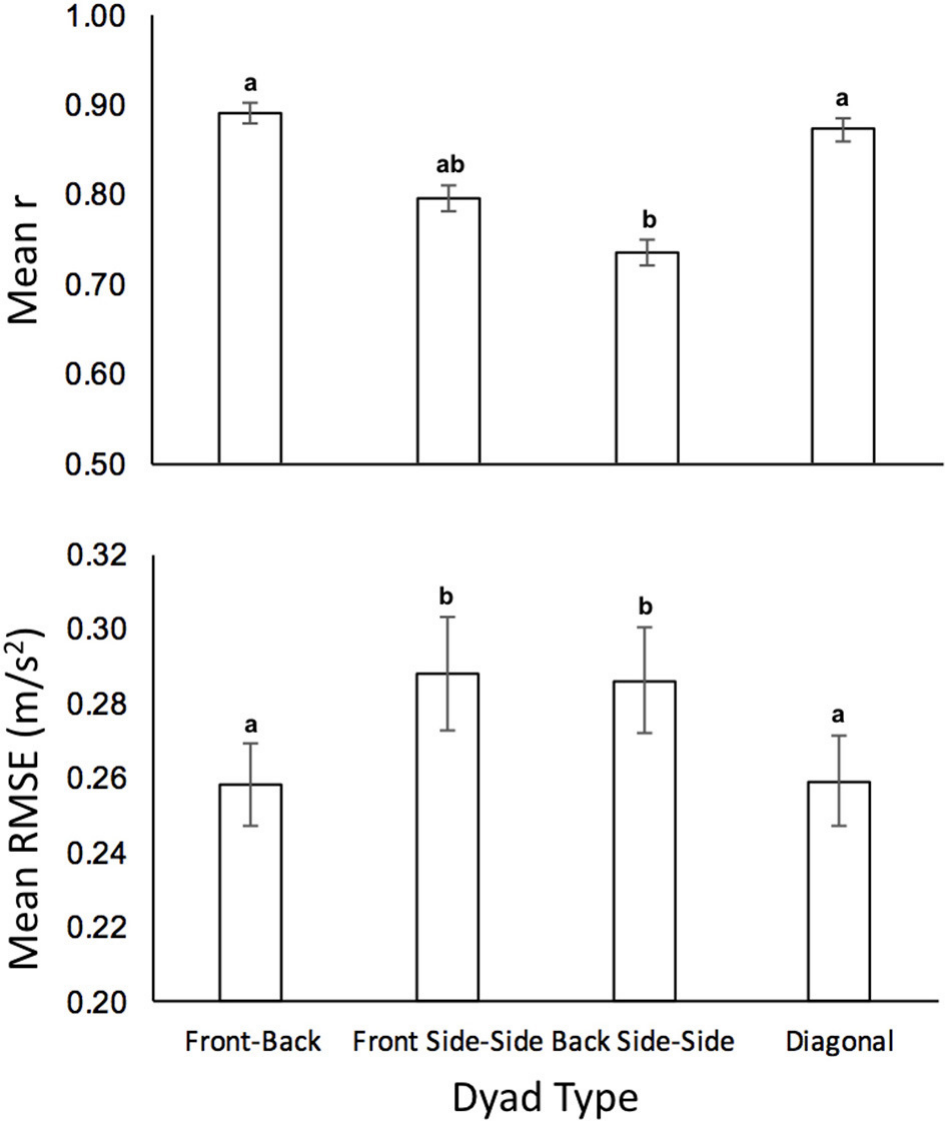
Bar graphs of simulation results for mean correlations between real dyads **(Top)**, and RMSE between real dyads **(Bottom)**. Duncan grouping specifies significant differences.

Sample time series of the simulated and observed “follower” acceleration (both in red), together with the observed “leader” acceleration (in blue), for four dyads appear in Figure 9. The mean correlation for the front-back dyads was *r* = 0.89 ± 0.33 (RMSE = 0.26 m/s^2^), for the diagonal dyads was *r* = 0.87 ± 0.01 (RMSE = 0.26 m/s^2^), for the front side-side dyad was *r* = 0.79 ± 0.30 (RMSE = 0.29 m/s^2^), and for the back side-side dyad was *r* = 0.74 ± 0.30 (RMSE = 0.28 m/s^2^; Figure 10 top). A two-way ANOVA on transformed *r* revealed a main effect of dyad, *F*_(3, 64)_ = 8.00, *p* < 0.001, η^2^ = 0.27. *Post-hoc* comparisons with Bonferroni correction indicated that the model performs significantly better on front-back dyads and diagonal dyads than on the back side-by-side dyad (*p* < 0.001 and *p* < 0.01, respectively), probably because back dyads are less strongly coupled to each other and influenced by the front dyad. *Post-hoc* comparisons with Bonferroni correction showed no significant pairwise differences in correlation (*p* > 0.05) as a function of density.

A similar pattern of results holds for statistical tests on RMSE of speed (see Figure 10, bottom). A two-way ANOVA revealed a main effect of dyad on RMSE, *F*_(3, 64)_ = 6.86, *p* < 0.001, η^2^ = 0.24, and a main effect of density, *F*_(3, 64)_ = 6.81, *p* < 0.001, η^2^ = 0.24, but no interaction, *F*_(6, 48)_ = 0.48, *p* > 0.05. Bonferroni-corrected *post-hoc* comparisons confirmed that the model performs better on front-back dyads and diagonal dyads than on both side-by-side dyads (*p* < 0.05).

In sum, the speed-matching model generalizes from pairs of pedestrians to small groups. It provides a close approximation of the local speed coupling, and successfully explains both pairwise coordination and an emergent group speed.

## Discussion

The present experiment investigated the degree of coordination in pedestrian groups during goal-directed walking, with the aim of analyzing the effects of a visual coupling, group density, and neighbor position on collective behavior. We analyzed the behavioral variables heading Φ and speed *s* in a four-pedestrian group, yielding an eight DoF system. We then submitted the behavioral variables to a global (collective) analysis: (1) PCA to index the dimensional compression of group behavior; and to local (pairwise) analyses: (2) linear cross-correlation to estimate the coupling strength between dyads in a group, and (3) non-linear CRQ to measure the dynamic stability of the local coupling.

Our main finding is that most analyses yielded evidence of spontaneous coordination in walking speed due to the visual coupling in real groups, compared to reshuffled virtual groups. It is important to point out that the external task constraints in this experiment (common goal, simultaneous go signal, simultaneous goal command, similar preferred walking speeds) by themselves induced similar behavior across individuals, which we estimated using the shuffled virtual groups. We expect that emergent heading and speed coordination would be observed in less restricted contexts, and research is under way to study spontaneous coordination in both heading and speed.

At the global level of analysis, the PCA indicated that visually coupled pedestrian groups exhibited significant dimensional compression across all experimental conditions. Note that the external task constraints accounted for a reduction of ~2.2 DoF (from 8 to 6.2) in the virtual groups, a 23% reduction in DoF. Yet the visual coupling produced a further reduction of ~2.6 DoF (from 6.2 to 3.6) in the real groups, or an additional 33% reduction in DoF. This is indicative of a functional reorganization of DoF via the informational coupling of behavioral variables, consistent with the emergence of collective coordination. These results are similar to those of Ramenzoni et al. (2012), who demonstrated dimensional compression in an interpersonal supra-postural task, and support the reduction of DoF in interpersonal coordination proposed by Riley et al. (2011).

The analysis of the composition of PC1 offers preliminary evidence of a new collective variable underlying the emergence of group coordination in the context of the current task. The loading of behavioral variables on PC1 suggests that speed coordination is a primary contributor to the collective behavior, whereas heading coordination was no greater in the real than the virtual group. Further, the analysis of the composition of PC2 demonstrated that the heading and speed loading is not simply dichotomous, as evidenced by the bimodal distribution for the heading coefficients in the real group (Figure 5B) and the negatively skewed unimodal distribution of the speed coefficients (Figures 6B). This indicates that the heading behavioral variable was a relatively weak contributor to the first two PCs overall. Thus, the remaining discussion focuses on the analysis and modeling of speed coordination.

At the local level of analysis, the cross-correlations for speed indicated a high visual coupling strength within the groups. Specifically, a significantly higher mean correlation was found for the real group (*r* = 0.84) compared to the virtual group (*r* = 0.36), independent of dyad. This can be explained similarly to the PCA results, in that the visual coupling increased the speed correlation for all dyads. It appears that local coupling strengths can be reliably estimated by pairwise linear correlations. However, the pairwise cross-correlations did not reveal a significant difference between types of dyads. This could be due, in part, to the possibility that back participants were influenced by more than one neighbor at a time. We are currently developing a neighborhood model that allows us to estimate the combined influence of multiple neighbors.

The non-linear CRQ analysis provided further evidence regarding the strength and stability of the local coupling. Speed coordination exhibited a longer CML in real groups than in virtual groups, indicating that the visual coupling was dynamically stable. Specifically, real dyads were stably coupled for almost two full seconds (i.e., 111.13 samples at 60 Hz), at some point in each 6–8 s trial.

Taken together, the PCA, cross-correlation, and CRQ results indicate that the global coordination in the present task is due in large part to the local coordination of speed, which in turn emerges from the visual coupling between individual pedestrians. Finally, we tested whether an empirical model of the local speed coupling could reproduce the observed coordination patterns. The simulation results supported this interpretation, for the coordination of dyads in a group is reproduced by the speed-matching model. The simulation results show that the speed-matching model generalizes from pairs of pedestrians to pedestrian groups, and imply that the local coupling is sufficient to explain the adoption of a common speed. We conclude that the local visual coupling can account for the pattern of global coordination.

Somewhat to our surprise, we did not observe a consistent effect of density on the degree of coordination. In fact, no measures yielded significant density effects, consistent with our previous finding that speed coordination in following is independent of interpersonal distance over 1–3 m (Rio et al., 2014). It is possible that the range of densities tested (0.5–2.5 m spacing) was insufficient to reveal an effect, or that the external task constraints, combined with a short walking distance, limited the degree of variation in the data. Research is in progress to test a wider range of densities (up to 4 m spacing) over longer walking distances, without a common goal or timing signals.

Finally, we would like to mention that we also performed an uncontrolled manifold (UCM) analysis on the eight-dimensional Φ and *s* data (Scholz and Schöner, 1999), as another way to estimate the reduction in effective DoF. This approach was unsuccessful, and it is instructive to consider why that was the case. A UCM analysis depends on the existence of reciprocal compensation between two or more behavioral variables in the system, which is considered a signature of motor synergies. But in retrospect, there is no reason to expect reciprocal compensation in collective group behavior: the acceleration of one agent would not be expected to produce a compensatory deceleration by a coupled agent to maintain the mean speed, but rather a coordinated acceleration; similarly, a change in heading direction by a subset of agents would not be expected to yield compensatory heading changes in the other direction, but a coordinated turn by the group. This observation suggests that reciprocal compensation may not be a general characteristic of all forms of interpersonal coordination in human groups (cf. Riley et al., 2011).

The present work is a starting point for understanding collective behavior in pedestrian groups. We began by analyzing the local coupling in dyads, on the hypothesis that this generic coordination mechanism would scale up to small groups, large crowds, and even flocks or schools in other species. Expanding the methodological framework of interpersonal coordination (Riley et al., 2011; Ramenzoni et al., 2012) to the behavior of small groups, we obtained evidence of dimensional compression and speed coupling. The present framework provides a foundation for the analysis and modeling of local and global coordination in future research. It is likely that other factors may also constrain group coordination. For example, cognitive processes such as decision-making and motivation, and social factors such as group membership, dominance relations, and social communication, may influence the selection of goals, neighbors, walking speeds, and control laws and shape the emergent crowd dynamics. The present experiment evaluates ways of quantifying local and global coordination in many of these contexts, and offers an approach to characterizing emergent collective behavior.

## Author contributions

AK led all behavioral analyses on the project, and was the lead contributor on the manuscript. KR and SB participated in the experimental design, led all data collection, data post-processing, modeling and simulation efforts. They also contributed to the write-up of the manuscript. AW contributed to the behavioral analyses and the write-up of the manuscript. WW was the leader on the project and provided guidance on the project method and analyses, and also provided support during write-up of the manuscript.

### Conflict of interest statement

The authors declare that the research was conducted in the absence of any commercial or financial relationships that could be construed as a potential conflict of interest.
